# Using artificial intelligence to improve public health: a narrative review

**DOI:** 10.3389/fpubh.2023.1196397

**Published:** 2023-10-26

**Authors:** David B. Olawade, Ojima J. Wada, Aanuoluwapo Clement David-Olawade, Edward Kunonga, Olawale Abaire, Jonathan Ling

**Affiliations:** ^1^Department of Allied and Public Health, School of Health, Sport and Bioscience, University of East London, London, United Kingdom; ^2^Division of Sustainable Development, Qatar Foundation, College of Science and Engineering, Hamad Bin Khalifa University, Doha, Qatar; ^3^Endoscopy Unit, NHS Trust, Epsom and St. Helier University Hospitals, Carshalton, United Kingdom; ^4^School of Health and Life Sciences, Teesside University, Middlesbrough, United Kingdom; ^5^Department of Biochemistry, Adekunle Ajasin University, Akungba-Akoko, Nigeria; ^6^Independent Researcher, Stockton-on-Tees, United Kingdom

**Keywords:** artificial intelligence (AI), public health, healthcare, review, health policy

## Abstract

Artificial intelligence (AI) is a rapidly evolving tool revolutionizing many aspects of healthcare. AI has been predominantly employed in medicine and healthcare administration. However, in public health, the widespread employment of AI only began recently, with the advent of COVID-19. This review examines the advances of AI in public health and the potential challenges that lie ahead. Some of the ways AI has aided public health delivery are via spatial modeling, risk prediction, misinformation control, public health surveillance, disease forecasting, pandemic/epidemic modeling, and health diagnosis. However, the implementation of AI in public health is not universal due to factors including limited infrastructure, lack of technical understanding, data paucity, and ethical/privacy issues.

## 1. Introduction

Artificial intelligence (AI) is a vast field that includes a variety of methodologies, such as computer vision, natural language processing, and machine learning (1). These methods are useful for pattern recognition, prediction, and large-scale data analysis. AI has the potential to be transformative within the healthcare domain. AI has been applied to disease diagnostics, helped forecast the development of infectious diseases, and find novel medication targets (2). AI has also been used to guide interpretation of medical imagining, and drug discovery and delivery (3). However, during the COVID-19 pandemic, there was a shift in the exploitation of AI from medicine to public health. During the pandemic, AI was integral to forecasting COVID-19 spread, contact tracing, pharmacovigilance, and rapid testing and detection (4, 5). The implementation of some of these epidemiology informatics tools aided global efforts to cut the spread of the COVID-19 virus and improved patient care. Table 1 highlights some examples of how AI was employed to curb the COVID-19 pandemic.

**Table 1:** AI-based measures to ameliorate the impact of the COVID-19 pandemic.

AI-aided response	Mechanism of action	References
COVID-19 diagnosis	Computer-aided diagnosis from CT images	(6)
COVID-19 outbreaks surveillance	Identify COVID-19 outbreaks from contact-tracing interview forms	(7)
Contact tracing	Mask wearers facial recognition surveillance	(8)
COVID-19 misinformation control	Use of social media companies like Facebook, Twitter, Instagram, TikTok, and LinkedIn to curb misinformation	(9, 10)
Automated COVID-19 screening	Attention-based deep 3D instance learning	(11)
Drug prediction	Identification of drugs that can be repurposed to fight COVID-19 virus	(12)
Drug formulation	Deep learning to develop drugs to combat COVID-19 virus	(13)
COVID-19 severity	Machine learning tools to predict COVID-19 severity	(14)
COVID-19 transmission prediction	Machine learning models to forecast COVID-19 spread	(15)
COVID-19 pharmacovigilance	Social media pharmacovigilance of COVID-19 vaccination to identify adverse effects	(16)
Surveillance using BlueDot	Prediction and spread of COVID-19 via air ticket sales, climate, and demographic data	(17)
Forecast using HealthMap	Forecast COVID-19 spread from social media and other web data	(17)
Reliable diagnosis using Infervision	CT scan screening for COVID-19	(17)

Other potential advantages of AI for public health including increased effectiveness, precision, and scalability of public health treatments. AI can also assist in locating novel insights and patterns that human analysts might miss (18). There are, however, significant ethical and regulatory concerns that must be addressed, such as data privacy and bias in AI systems. In addition, the exploitation of AI to improve public health is not spread evenly across the globe. Some of the major factors that potentially impede the advancement of AI in public health are data availability/governance, availability of related infrastructure, technical skills gap, and the possibility of inequity/bias due to data discrepancies (19). To ensure that the technology is used ethically and responsibly, researchers, healthcare practitioners, and policymakers must work together to integrate AI into healthcare systems. This article reviews recent trends in AI for public health and considers both the potential benefits and challenges of this technology.

## 2. Methods

This study employed a PRISMA (Preferred Reporting Items for Systematic Reviews and Meta-Analyses) Scoping Review approach to comprehensively explore the application of AI to improve public health (20, 21). Relevant electronic databases (e.g., PubMed, Scopus, Web of Science) were carefully searched using appropriate keywords and controlled vocabulary terms related to AI and public health. Initial screening of titles and abstracts was conducted to identify potentially relevant studies. Full-text articles of the selected studies were retrieved and evaluated for eligibility based on the predefined inclusion and exclusion criteria. The inclusion criteria encompassed studies that focused on the application of AI in public health and were published in English. Exclusion criteria included studies unrelated to public health, studies published in languages other than English, and studies lacking full-text availability. Findings were synthesized and presented in a narrative format identifying key themes, trends, and patterns related to the application of AI to improve public health. Also, we discussed the implications of these findings for public health practice.

### 2.1. History of AI in public health

The 1960s saw the beginning of artificial intelligence (AI) research, which first aimed to create systems that could mimic human intelligence (22). Expert systems, which utilized knowledge from human specialists to give decision assistance for medical diagnosis and treatment planning, were the main focus of early AI applications in healthcare. Expert systems were still the main focus of AI research in the healthcare sector in the 1980s and 1990s, but machine learning and natural language processing were also being investigated (23). Researchers were able to begin exploring the potential of AI in domains such as medical diagnosis, drug discovery, and public health surveillance as a result of the availability of enormous databases of medical information and sophisticated computer systems.

The 2000s saw advancements in computer vision, natural language processing, and machine learning that made it possible for researchers to create increasingly complex AI systems that could evaluate vast amounts of data and anticipate future outcomes (24). Due to this, AI-based diagnostic systems were created, such as those that analyze medical images and help with the identification of diseases like cancer. Additionally, improvements in text mining and natural language processing methods allowed academics to use AI to evaluate vast amounts of unstructured data, such as electronic health records, and derive insightful conclusions.

Recent years have seen a rise in interest in the use of AI within public health, notably in the fields of predictive modeling and public health surveillance. AI algorithms, for instance, have been used to forecast the spread of infectious diseases like COVID-19 or influenza (25), enabling public health officials to take preventive measures. They have also been used to analyze vast amounts of data from social media and other sources to spot potential outbreaks and monitor the spread of diseases. Furthermore, the application of AI in public health has grown to include new fields like drug development and personalized treatment (26) due to the increased accessibility of massive data and sophisticated computing resources. In general, there has been a growth in the history of AI in public health from primitive expert systems to more advanced systems that can examine vast volumes of data and make predictions. The use of AI in healthcare has a number of potential advantages for public health, but there are also ethical and legal concerns such as data privacy and surveillance, safety, transparency, fairness and biases of algorithms, as well as prospective philosophical conundrum of the function of human judgment (27–29) that must be taken into account in order to integrate this technology into healthcare systems.

### 2.2. Predictive modeling

**Figure 1:**
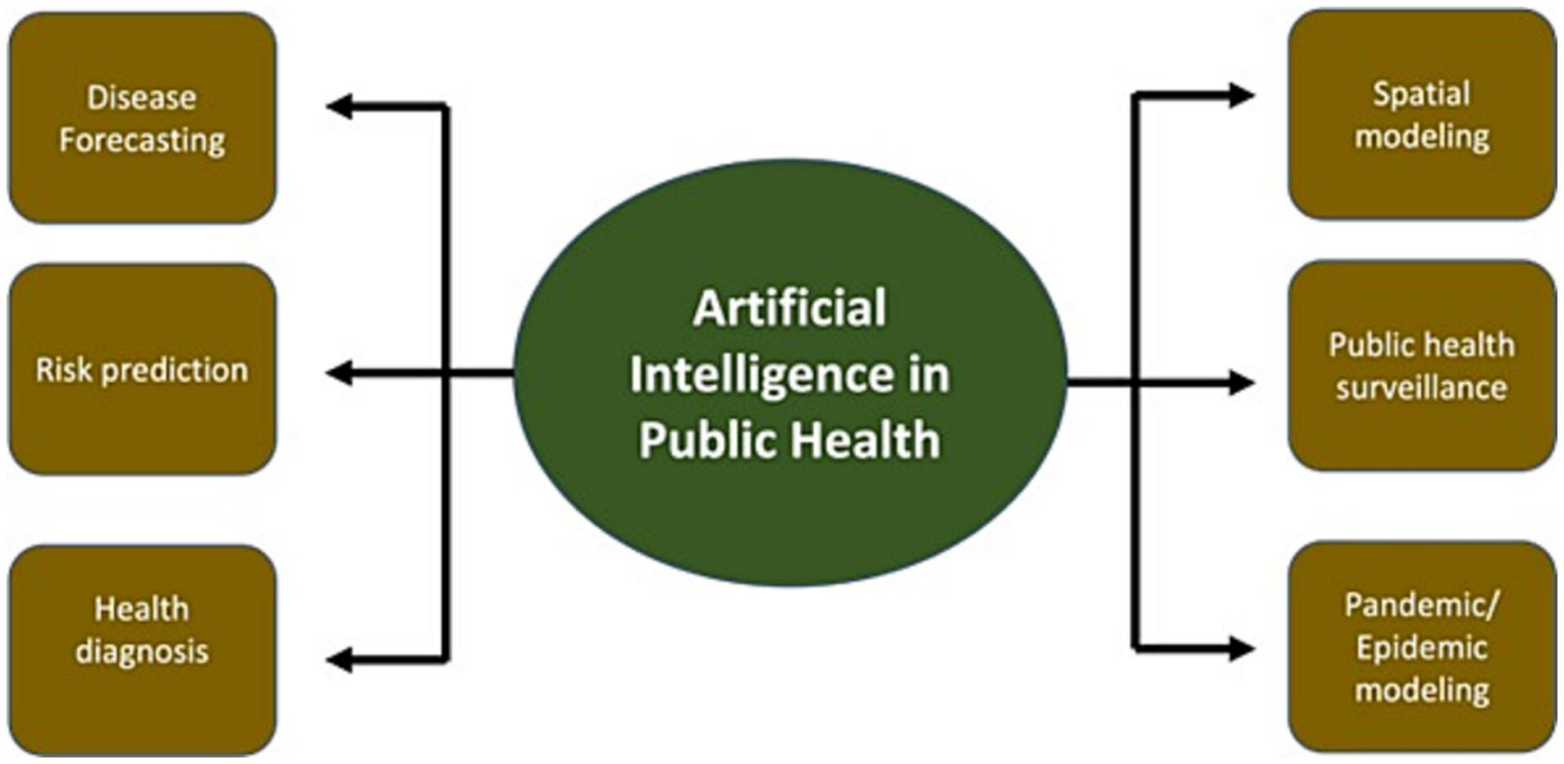
Predictive modeling of AI in public health.

In order to examine data and forecast future outcomes, predictive modeling combines statistical models and machine learning techniques. Predictive modeling has been used in public health to foresee the spread of infectious diseases like COVID-19 and influenza (30, 31). Predictive models can find patterns and trends that can guide public health measures by examining data on previous epidemics and other pertinent elements, such as population demographics and weather patterns (32). As predictive modeling has the potential to enhance our capacity to forecast the spread of infectious illnesses and guide public health treatments, predictive modeling is a key application of AI for public health (25). Figure 1 illustrates a predictive modeling framework of AI in public health.

The core problem to be solved by the use of AI for predictive modeling, encompassing disease forecasting, risk prediction, and spatial modeling, is the enhancement of accuracy, efficiency, and actionable insights in public health decision-making (33, 34). Traditional methods in these domains often face limitations in handling the complexity of data, identifying patterns, and making accurate predictions. This is where AI emerges as a transformative solution to address these challenges and achieve more effective outcomes.

The absence of clear categorization and summarization of traditional and AI methods for predictive modeling, such as disease forecasting, risk prediction, and spatial modeling, hinders informed decision-making, efficiency, accessibility, and research collaboration in the field of public health. A lack of structured classification makes method selection challenging, delays implementation, and deters broader adoption. Developing a standardized taxonomy and concise summaries for each approach is crucial to enhance the field’s progress, enabling practitioners to navigate methods efficiently, accelerate decision-making, and facilitate research collaboration (35, 36).

#### 2.2.1. Disease forecasting

Disease forecasting is an important application of (AI in public health) (37), as it has the potential to enhance our ability to anticipate the spread of infectious illnesses and subsequently inform and direct public health measures. This is a crucial component of public health because it enables officials to prevent outbreaks and act rapidly if they do occur. Historically, time-series analysis and other conventional statistical techniques were used in illness forecasting (38). However, with the development of AI, it is now possible to utilize more complex algorithms and evaluate a greater variety of data to produce more accurate predictions. The application of machine learning algorithms is one of the major trends in AI for disease prediction (39, 40). These algorithms may examine a variety of data sources, including social media and electronic health records, to find patterns and forecast the spread of diseases (41).

Another development in AI for illness forecasting is the expanding accessibility of huge data and cutting-edge computer resources (42). This makes it possible to analyze massive and varied data sets, like electronic health records, social media, and sensor data, in order to more accurately anticipate the future and spot patterns that were previously challenging to spot. The ability to evaluate vast volumes of data, identify patterns and trends, and estimate future results are some of the potential advantages of AI for disease forecasting in public health. This can serve to guide public health initiatives and stop or reduce the spread of infectious diseases. Furthermore, the application of AI to disease forecasting can boost prediction effectiveness and precision, which could ultimately result in better health outcomes for individuals and communities. However, there are limitations to utilizing AI in public health for illness forecasting (43). As the accuracy of predictions depends on the quality and completeness of the data used to train the algorithms, finding high-quality data is a key challenge. Furthermore, when applying AI in public health, there are also ethical and legal considerations to be made, notably in regards to data security and privacy.

The use of AI in public health for illness forecasting is anticipated to develop further. Integration of AI with other technologies, such as the Internet of Things (IoT) and wearable devices, which may give real-time data and increase the accuracy and timeliness of predictions, is one area that has the potential to flourish. Additionally, explainable AI (XAI) techniques are being developed, which can increase the accountability and transparency of AI-based illness forecasting systems by revealing how algorithms produce predictions. The application of AI for personalized disease forecasting, where algorithms may examine information from electronic health records and other sources to predict the risk of disease for particular patients and guide treatment decisions, is another field with potential growth. Additionally, combining spatial data with GIS (Geographic Information System) technology can improve local level predictions and guide focused interventions in disease forecasting (44).

The core problem in disease forecasting is to predict the future spread and impact of diseases accurately. Traditional methods based on historical data and statistical techniques may struggle to capture complex dynamics and evolving patterns. AI, particularly machine learning and deep learning algorithms, addresses this issue by efficiently analyzing large datasets, identifying hidden relationships, and detecting intricate trends (36). The aim is to provide early warnings, actionable insights, and strategies for mitigating disease outbreaks. AI is being used to forecast the spread of diseases such as COVID-19. For example, Google AI has developed a model that can predict the number of COVID-19 cases in a given region up to two weeks in advance (45, 46).

#### 2.2.2. Risk prediction

A crucial component of public health is risk prediction because it enables focused disease management or prevention actions. Traditional risk prediction techniques, such as manual computations based on clinical and demographic data, can take time and may not always yield reliable findings (47). AI has the potential to increase the effectiveness and precision of risk predictions, resulting in better results for public health. Large amounts of data, like electronic health records, can be analyzed by machine learning algorithms to find patterns and predict the likelihood of diseases. Furthermore, these algorithms may examine intricate data, including genomics and medical images, to find patterns that can assess the likelihood of a disease.

AI risk prediction in public health is likely to develop further. Integration of AI with other technologies, including wearable gadgets and genomics, has the potential to deliver more accurate predictions by supplying more precise and real-time data. Additionally, explainable AI (XAI) tools can help increase the accountability and openness of AI-based systems by revealing how algorithms make predictions, and so promote trust in the use of AI in healthcare.

For risk prediction, the core problem is to identify individuals who are at an elevated risk of developing specific diseases. Traditional approaches rely on demographic and clinical data analysis, which may not fully capture subtle risk factors or evolving conditions. AI methods, including machine learning and natural language processing, enhance risk prediction by integrating diverse data sources, detecting non-linear relationships, and identifying latent patterns (48). The objective is to tailor interventions, allocate resources, and improve personalized healthcare strategies. AI is being used to predict the risk of events such as heart attacks, strokes, and car accidents. For example, IBM Watson Health has developed a model that can predict the risk of heart attack with 90% accuracy (49).

#### 2.2.3. Spatial modeling

Spatial modeling—analyzing geographic information to recognize patterns and trends in health outcomes—is an essential component of public health as it enables the localization of interventions to areas with the highest burden of disease. Conventional spatial modeling techniques, such as manual data gathering and analysis, may not always produce accurate findings and can take a long time to complete (50). AI can increase the effectiveness and precision of geographical modeling, improving public health outcomes.

Large-scale geographic data, like satellite images, can be analyzed by machine learning algorithms to find trends and forecast where diseases will spread. For instance, such techniques have been used to forecast the risk of dengue fever, including dengue cases, rate, peak time, and peak intensity, as well as dengue risk predictors, including mosquito biting rate (51–53). Integration of geographic information systems (GIS) with AI is a further development in spatial modeling in public health. This enables the examination of massive and varied data sets, such as social media data and electronic health records, in a geographical context to generate more accurate forecasts and identify trends that were previously challenging to spot. Using deep learning algorithms for spatial modeling in public health is another emerging trend in AI. These algorithms can examine complicated data, including genetics and medical images, to find patterns that can signal the danger of disease in specific regions. For example, one study used this to consider how certain particular brain regions are connected to specific neurological disorders (54). Other work study used it to improve diagnosis of respiratory disorders by taking audio recording of patients’ coughs in addition to symptom reports (55).

Gunasekeran et al. (56) conducted a systematic scoping review focusing on digital health applications for public health responses to COVID-19. Their review emphasizes the role of AI in predictive modeling. By leveraging AI algorithms, predictive models can analyze vast amounts of data, including demographics, health records, and environmental factors. These models enable the forecast of disease spread, identification of high-risk populations, and the development of targeted interventions.

In spatial modeling, the core problem revolves around uncovering geographical patterns and trends in health outcomes. Traditional methods often lack the ability to handle the complexity of spatial data, identify interactions, and make accurate predictions at local levels. AI, coupled with Geographic Information Systems (GIS), offers solutions by utilizing machine learning and deep learning techniques. This enables the identification of intricate spatial patterns, such as disease clusters, and supports data-driven decision-making for targeted interventions (Bolus et al., 2019). AI is being used to model the spread of diseases and other phenomena across space. For example, the University of California, Berkeley has developed a model that can predict the spread of wildfires (57).

### 2.3. Electronic health records

Research and practice in public health benefit greatly from the information contained in electronic health records (EHRs). Digital records of patient health information, such as medical history, prescription use, lab results, and other pertinent information, are kept in EHRs. They are more common in healthcare settings but offer a plethora of information for research and practice in public health. However, the sheer amount of data in EHRs can make manual analysis difficult, necessitating the development of new technology to draw conclusions from the data. AI has the ability to enhance the effectiveness and precision of EHR data processing, improving the outcomes for public health. For example, for extracting patient clinical data, such as vital signs, laboratory results, and drug prescriptions (58, 59).

The application of machine learning algorithms is one of the major themes in AI for EHRs in public health. These algorithms can analyze large quantities of data, like electronic health records, and find trends and anticipate how diseases will spread. Natural language processing (NLP) methods are another AI development for EHRs in public health. These methods can extract data from unstructured text sources, like doctor’s notes, to better comprehend the health status of a patient. EHR analysis is now increasingly using deep learning algorithms, which can evaluate complex data and produce highly accurate predictions. These formulas have been applied to forecast patient outcomes, including readmissions to hospitals, and can be used to help develop public health policy, such as through ascertaining whether specific population groups would benefit from targeted interventions (such as vaccinations for groups more vulnerable to specific vaccine-preventable diseases). The use of AI to EHRs can boost prediction effectiveness and precision, which could ultimately result in improved health outcomes for both individuals and communities.

AI algorithms are capable of processing large volumes of EHR data to extract valuable insights. These insights aid in the identification of disease patterns, personalized treatment approaches, and early detection of outbreaks. By leveraging AI in EHR analysis, healthcare professionals can make more informed decisions and deliver optimized care (56).

The application of AI for EHRs in public health is not without its challenges, though. As the accuracy of predictions depends on the quality and completeness of the data used to train the algorithms, finding high-quality data is one a key issue. It is essential to protect the security and privacy of patient data, and the use of AI on EHRs must adhere to laws like the Health Insurance Portability and Accountability Act (HIPAA) in the US. Lack of uniformity in EHR systems can also make it challenging to interpret data from various sources. Additionally, it may be challenging to construct reliable AI algorithms for analysis because of the depth and diversity of the data in EHRs, which includes unstructured text, photos, and time series data.

Future predictions call for AI for EHRs in the public health sector to develop further and reach new frontiers (60). Using AI for personalized medicine, where algorithms may examine information from electronic health records and other sources to forecast the risk of disease and guide treatment decisions for specific patients, is one field with promise for growth.

AI for EHRs in public health has the potential to increase data analysis’s effectiveness and accuracy, resulting in better public health outcomes. While difficulties remain with using AI in public health, such as the requirement for high-quality data and ethical issues, there are also many potential advantages to this technology. Future studies in this field should concentrate on improving the precision and efficiency of algorithms, addressing moral and legal concerns, and standardizing EHR platforms.

Research involving Electronic Health Records (EHRs) combined with Natural Language Processing (NLP) and Artificial Intelligence (AI) techniques has gained traction in recent years, aiming to extract valuable insights from the unstructured textual data within EHRs. NLP methods encompass techniques like Named Entity Recognition (NER) to identify medical terms, sentiment analysis for patient feedback, and text classification for diagnoses (61). AI methods, such as machine learning and deep learning, are employed to predict disease outcomes, recommend treatments, and enable personalized medicine by integrating structured EHR data with NLP-processed textual information. In particular, deep learning models like recurrent neural networks (RNNs) and transformer-based architectures (e.g., BERT) excel in handling sequential data and capturing intricate contextual relationships within EHR narratives (62, 63). These models facilitate accurate information extraction and semantic understanding of medical text, which is essential for meaningful analysis.

Table 2 captures recommended listing of public databases on EHRs. These public databases provide a foundation for researchers to apply NLP and AI techniques to EHRs, enabling advancements in disease prediction, treatment recommendations, patient outcome analysis, and more. However, ethical considerations and data privacy must be upheld when working with EHRs, ensuring the secure handling of sensitive patient information.

**Table 2:** Recommended listing of public databases on EHRs.

Public databases on EHRs	Details
MIMIC-III (Medical Information Mart for Intensive Care III)	A freely accessible database containing de-identified EHRs of patients admitted to intensive care units, including clinical notes, vital signs, medications, and more.
eICU Collaborative Research Database	Offers EHR data from patients in critical care units across multiple healthcare institutions, enabling researchers to study diverse patient populations.
National Inpatient Sample (NIS)	Part of the Healthcare Cost and Utilization Project (HCUP), providing information on inpatient stays and EHR data across a wide range of hospitals.
NLP Research Datasets	Datasets like i2b2, n2c2, and MedNLI offer labeled EHR data for specific NLP tasks, encouraging the development and benchmarking of AI models.
Observational Health Data Sciences and Informatics (OHDSI)	A collaborative effort providing access to real-world EHR data for observational research, allowing AI-driven insights into various diseases and treatments.
HealthData.gov	Hosts a variety of datasets related to health, including EHRs, offering opportunities for researchers to explore different facets of healthcare using AI methods.
Clinical Data Integration and Sharing (CDIS)	An initiative by the National Institutes of Health (NIH) offering EHR data for research purposes, promoting interdisciplinary studies in healthcare.

## 3. Diagnostics

Diagnosis is a crucial component of public health because rapid and precise disease diagnosis is necessary for efficient disease treatment and management. Traditional diagnostic techniques, such as laboratory testing, can be expensive and time-consuming, and their results may not always be reliable. AI has the ability to increase the speed and precision of diagnostic procedures, improving the outcomes for public health.

Machine learning algorithms can examine and integrate massive volumes of data, including laboratory test results and medical imaging, to find patterns and forecast disease. Deep learning algorithms, which can evaluate complex data and produce predictions with high accuracy, are especially helpful for deciphering patterns in medical pictures like x-rays and CT scans that could point to the presence of disease. Using NLP methods can also be used to extract data from unstructured medical text, such as doctor notes and medical reports, to reveal data patterns that may predict disease.

AI can enhance the speed and precision of diagnostic procedures, improving both the health of the individual and the community as a whole (64). The cost of lab tests and other diagnostic procedures may also be decreased by using AI in diagnostics, with human expert oversight. The application of AI for diagnostics in public health is not without its drawbacks. As the accuracy of predictions depends on the quality and completeness of the data used to train the algorithms, finding high-quality data can be challenging. Furthermore, performance tests, validation, and comparisons with conventional diagnostic techniques should be compared to those drawn from AI-based diagnostic systems. Another difficulty is the requirement for a sizable volume of labeled data, which might not always be available. Doctors may also require training to comprehend how AI-based diagnostic algorithms derive their results to explain their forecasts to patients or policymakers.

Medical diagnosis has witnessed a transformative shift with the integration of AI methods, such as Convolutional Neural Networks (CNNs), Transformer-based models, NLP-based approaches, and more, in analyzing diverse data modalities like ultrasound, X-ray, CT, MRI, and physiological images. This convergence of advanced AI techniques and multi-modal medical data has significantly enhanced diagnostic accuracy, speed, and personalized treatment recommendations (56).

CNN-based methods excel in image analysis and have revolutionized medical imaging diagnostics. By automatically learning hierarchical features from images, CNNs enable the identification of patterns, anomalies, and abnormalities in X-rays, CT scans, and MRIs (65). The ability to capture spatial relationships within images has enabled improved disease detection, localization, and classification. Originally designed for natural language processing, transformer-based models like BERT and its variants have been adapted for medical diagnosis (63). These models excel in capturing contextual relationships, enabling comprehensive understanding of medical text reports, clinical notes, and radiology reports. Their application enhances decision-making and assists in diagnosing complex conditions. NLP-based methods play a crucial role in extracting valuable insights from textual data in medical records, patient histories, and research articles. Named Entity Recognition (NER) and sentiment analysis aid in understanding patient experiences and identifying critical medical terms, facilitating accurate diagnosis and treatment recommendations.

The use of AI in public health diagnostics has the potential to increase the speed and precision of diagnostic procedures, improving the outcomes for population health. While there are certain limitations with using AI in public health, such as the requirement for high-quality data and ethical issues, there are also many potential advantages to this technology. Future research in this field should concentrate on creating more precise and effective algorithms, resolving moral and legal concerns, and expanding the accessibility of labeled data for AI model training. The development of explicable AI methodologies for diagnostic systems may contribute to boosting public confidence in the use of AI in healthcare and for the development of public health policy, and in enhancing the systems’ accuracy.

### 3.1. Public health surveillance

Public health surveillance has always relied on labor-intensive, error-prone manual data gathering and interpretation (66). AI has become a potent tool for public health surveillance (67, 68) due to the rise in the amount of health-related data being produced, including that from electronic health records (EHRs), social media, and sensor data (69, 70). In comparison to conventional approaches, AI systems can evaluate vast amounts of data and more rapidly and identify trends and give advance warning of potential disease outbreaks and epidemics (71).

The Centers for Disease Control and Prevention (CDC) is using AI to track the spread of COVID-19. The CDC has developed a system that uses AI to analyze data from a variety of sources, including electronic health records, social media, and travel data. This system can be used to identify potential outbreaks and to track the spread of the virus in real time (72, 73).

Infodemiology and infoveillance, informed by AI and data mining techniques, allow for the analysis of search behavior, communication patterns on social media, and publication trends. These AI-driven methods provide real-time insights into disease trends, public sentiments, and misinformation. Public health authorities can leverage this information for early detection, response planning, and effective communication strategies (74–76).

The ability to evaluate massive amounts of data, recognize patterns and trends, and forecast are some of the potential advantages of AI for public health surveillance (49, 77). This can serve to guide public health initiatives and stop or reduce the spread of infectious illnesses (23). Furthermore, applying AI to public health surveillance can boost prediction effectiveness and precision (78), which could ultimately result in better health outcomes for both individuals and communities. As data for public health surveillance frequently come from numerous sources and may have different formats, integration and management of the data can be complex (79–81). The accuracy of predictions may suffer as a result since it may be challenging to combine the data and verify its consistency. Additionally, issues with bias and discrimination may arise from the application of AI in public health surveillance (82, 83). For instance, if the data used to train algorithms are skewed, the algorithm’s predictions are likely to also be skewed. This is especially problematic for public health surveillance (84), as inaccurate projections can result in the distribution of resources inequitably and have detrimental effects on marginalized communities.

### 3.2. Public health challenges of AI

To enable the secure and successful application of this AI in public health, several difficulties must be resolved. These include ethical and legal concerns, particularly in relation to data security and privacy (26, 68, 85). While HIPAA in the United States establishes guidelines for the use and distribution of protected health information (PHI) (86), with similar guidelines in operation in the European Union (General Data Protection Regulation, GDPR) and the UK (Data Protection Act 2018) with ensuring data privacy is crucial in establishing trust in the use of AI to support development of healthcare policy and practice (87). The ethical implications of concerns including bias and discrimination in AI-based systems, which can disproportionately harm vulnerable people, are also a concern.

The accuracy and efficiency of AI-based systems, on the other hand, are reliant on the precision and completeness of the data used to train the algorithms (88). The absence of transparency and comprehensibility in AI-based systems is one of the primary issues facing public health applications of AI. Policymakers may find it challenging to comprehend how the algorithm came to its conclusions as a result (89, 90), and this could also foster skepticism about the use of AI in the public health (91, 92).

Nonetheless, several guidelines emerged from COVID-19 that could be considered as a resource for the application of AI for public health. Such guidelines include, the WHO Pillars document (93) for operational planning and guidance which provides a framework for countries to prepare for and respond to a public health emergency. This includes several sections on the use of AI for public health. These include Pillar 1: Country-level coordination, planning, and monitoring, which proposes the use of AI to track the spread of disease, identify potential outbreaks, and coordinate the response to a public health emergency. Pillar 2 focuses on surveillance, rapid response teams, and case investigation explores the use of AI to collect data on disease outbreaks, identify and investigate cases, and track the effectiveness of interventions.

Similar considerations were explored at The AI for Health Global Summit in 2021. The summit produced a number of recommendations for the use of AI for public health, including building capacity for the use of AI in public health and promoting ethical and responsible use of AI in public health. Such strategies should be aligned with the WHO pillars document and should be based on evidence-based research. These are just a few of the many guidelines that have emerged from COVID-19 that could be considered as a resource for the application of AI for public health. These guidelines provide a framework for countries to use to prepare for and respond to a public health emergency and they highlight the potential of AI to improve public health.

Addressing data security and privacy protection is paramount for the successful application of AI in public health. While the use of patient data from multiple centers is unavoidable for robust and generalizable AI models, ensuring patient privacy and data security remains critical. Collaborative efforts involving data collaboration and sharing through techniques like federated learning offer potential solutions to these challenges (94, 95). The utilization of patient data from multiple centers enhances the diversity and comprehensiveness of AI models, resulting in more accurate and adaptable algorithms. However, this practice must be accompanied by rigorous data de-identification, aggregation, and anonymization protocols to safeguard patient privacy. Compliance with regulations such as HIPAA (Health Insurance Portability and Accountability Act) ensures that sensitive patient information is appropriately protected (86). Data collaboration and sharing enable the pooling of insights from multiple sources without centralizing sensitive data. Federated learning, a decentralized approach, allows AI models to be trained across various centers without sharing raw data. Instead, models are collaboratively updated using locally stored data, minimizing privacy risks. This technique ensures that data remains within its source institution while contributing to the development of a powerful, collective AI model (95). Despite its benefits, federated learning faces challenges, such as dealing with heterogeneity across datasets, communication inefficiencies, and ensuring model convergence. Researchers are actively working to overcome these hurdles through algorithmic advancements.

## 4. Implications of AI for development of public health policy

AI can be a valuable tool for the development of public health policy. The ability to draw together various datasets allows insights to be made that would otherwise be difficult to develop with traditional methods. Algorithms can be employed in an iterative manner, with results of policies monitored, and subsequently informing and improving future policies. Leveraging these insights will allow policies to be developed that are better-targeted, impactful and timely. AI has a great deal to offer policymakers but, like all new technologies, trust and education in how to use it effectively and responsibly are critical to its future uptake and usefulness.

## Author contributions

DO: conceptualization, methodology, literature search, writing—review and editing, formal analysis, writing—original draft, data curation, and project administration. OW: project administration, methodology, and search strategy. AD-O: methodology, writing—review and editing, and writing—original draft. EK: methodology, writing—review and editing, and data curation. OA: methodology, writing—review and editing, and formal analysis. JL: methodology, project administration, writing—review and editing, and supervision. All authors contributed to the article and approved the submitted version.
